# TGFβ activity released from platelet-rich fibrin adsorbs to titanium surface and collagen membranes

**DOI:** 10.1038/s41598-020-67167-3

**Published:** 2020-06-23

**Authors:** Francesca Di Summa, Zahra Kargarpour, Jila Nasirzade, Alexandra Stähli, Goran Mitulović, Tanja Panić-Janković, Veronika Koller, Cosima Kaltenbach, Heinz Müller, Layla Panahipour, Reinhard Gruber, Franz-Josef Strauss

**Affiliations:** 1 Department of Oral Biology, Medical University of Vienna, Vienna, Austria; 2 Department of Periodontology, School of Dental Medicine, University of Bern, Bern, Switzerland; 3 Core Facility Proteomics, Clinical Institute of Laboratory Medicine, Medical University of Vienna, Vienna, Austria; 4 Austrian Cluster for Tissue Regeneration, Medical University of Vienna, Vienna, Austria; 5 Department of Conservative Dentistry, School of Dentistry, University of Chile, Santiago, Chile; 6 Clinic of Reconstructive Dentistry, University of Zurich, Zurich, Switzerland

**Keywords:** Cell biology, Computational biology and bioinformatics

## Abstract

Platelet-rich fibrin (PRF) contains a broad spectrum of bioactive molecules that can trigger several cellular responses. However, these molecules along with their upstream responses remain mostly uninvestigated. By means of proteomics we revealed that PRF lysates contain more than 650 proteins, being TGF**-**β one of the few growth factors found. To uncover the major target genes regulated by PRF lysates, gingival fibroblasts were exposed to lysates obtained from PRF membranes followed by a whole genome array. We identified 51 genes strongly regulated by PRF including IL11, NOX4 and PRG4 which are characteristic TGF**-**β target genes. RT-PCR and immunoassay analysis confirmed the TGF**-**β receptor I kinase-dependent increased expression of IL11, NOX4 and PRG4. The PRF-derived TGF**-**β activity was verified by the translocation of Smad2/3 into the nucleus along with the increased phosphorylation of Smad3. Considering that PRF is clinically used in combination with dental implants and collagen membranes, we showed here that PRF-derived TGF**-**β activity adsorbs to titanium implants and collagen membranes indicated by the changes in gene expression and immunoassay analysis. Our study points towards TGF**-**β as major target of PRF and suggest that TGF**-**β activity released by PRF adsorbs to titanium surface and collagen membranes

## Introduction

Platelet-rich fibrin (PRF) has been proposed as an alternative approach to the application of recombinant growth factors to enhance wound healing and bone regeneration^1^. PRF is obtained by centrifugation and spontaneous coagulation of blood followed by the removal of the red corpuscle base^2^. The coagulated plasma contains a complex mixture of growth factors and other bioactive molecules enmeshed within a fibrin network^3,4^. This coagulated plasma can be further processed by squeezing out the serum, obtaining a PRF membrane. PRF membranes have become an attractive strategy to maximize the clinical outcomes by delivering growth factors at the surgical site, either alone or in combination with dental implants and collagen membranes^5,6^. For example, PRF membranes can preserve the alveolar ridge dimension following tooth extraction^7^. Furthermore, dental implants coated with PRF increase their stability during the early phases of osseointegration^5,8^. Additionally, when PRF is combined with a collagen membrane in a guided bone regeneration approach it can preserve the alveolar ridge profile^9^. However, and despite these promising clinical results, the underlying cellular and molecular mechanisms induced by PRF are poorly understood^10^.

Mesenchymal lineage cells are among the possible targets at sites where PRF is applied. In the oral cavity, mesenchymal cells are found in the gingiva^11^. Consequently, it is not surprising that gingival fibroblasts are common targets for assessment of cell responses. For example, cell proliferation or osteogenic differentiation in response to PRF can be measured via changes in gene expression^12,13^. This screening approach can be further refined by means of whole genome arrays. Gene arrays are comprehensive analytical tools and have been used to screen target genes that are regulated in monocytes in response to platelet-derived microvesicles^14^. Hence, the first aim of this study was to identify clusters of cellular responses that may help to find the upstream signalling pathways triggered by PRF since the genetic signature in oral fibroblasts exposed to PRF lysates has not yet been identified.

PRF lysates encompassing growth factors and other bioactive molecules that are released at the surgical site can be prepared by the freeze-thawing of PRF membranes^15^. Immunoassays have identified characteristic platelet-released molecules including TGF-β in PRF lysates^16^ and PRF-conditioned medium^17^. While advanced proteomic technology has been steadily refined to elucidate the protein composition of purified platelets^18^, this approach has not been utilized in characterizing the protein signature of PRF membranes. Thus, the relevance of proteomics technology is to extend current knowledge of growth factors and other proteins that may cause a potent cellular response. The second aim of the present study was to combine the proteomic approach with the findings from the gene array to understand which PRF-derived growth factors cause the major changes in gene expression.

PRF-derived growth factors such as TGF-β have been identified to adsorb to biomaterials including titanium^19,20^ and collagen membranes^21,22^. The biological role of this binding nevertheless remains to be elucidated. Thus, and since PRF is frequently combined with the aforementioned biomaterials, the question arises whether PRF-derived growth factors that cause the major changes in gene expression – adsorb to these biomaterials. We have therefore included a series of experiments, similar to our previous research with acid bone lysates^20,23^. In order to mimic a clinical situation in an *in vitro* setting, we examined whether the growth factors released by PRF activates mesenchymal cells bound to the respective biomaterials. This is clinically relevant as local application of recombinant TGF-β in a collagen sponge can enhance bone regeneration of rabbit skull defects^24^. Thus, the final aim of this research was to investigate if the growth factors, which cause the most robust gene expression changes, adsorb to titanium and collagen membranes.

In the present study we show that (i) TGF**-**β is found in PRF lysates based on proteomic analysis; (ii) PRF lysates provoke a robust activation of TGF**-**β signalling in oral gingival fibroblasts based on genomic screening and a series of specific downstream analysis; (iii) PRF-derived TGF**-**β activity adsorbs to titanium implants and collagen membranes.

## Results

### Proteomics analysis of PRF lysates

To understand the composition of the PRF lysates, a proteomic analysis was performed. Mass spectrometry revealed 652 proteins (Supplement Table 1). Interestingly, only a few classical growth factors were detected including TGF-β, hepatoma-derived growth factor (HDGF), and myeloid-derived growth factor (MYDGF). Identified were also the latent-TGF-β-binding protein 1 (LTBP1), hepatocyte growth factor activator (HGFAC), hepatocyte growth factor-like protein (MST1), epidermal growth factor receptor substrate 15 (EPS15), insulin-like growth factor-binding protein complex acid labile subunit (IGFALS). We could also confirm the presence of typical platelets proteins, such as platelet factor 4 (PF4), platelet basic protein (PPBP), platelets glycoproteins (GP1BA, GP1BB CD36, GP5, GP6, GP9), platelet endothelial cell adhesion molecule (PECAM1), and von Willebrand factor (VWF).

**Table 1:** Up-regulated genes with at least 5x changes in oral fibroblasts exposed to PRF lysates.

Gene Symbol	Fold Change
NOX4	12.1
MCM10	10.2
TSPAN13	9.7
FAM111B	9.0
ANGPTL4	8.6
MT1L	8.6
KCNN4	8.6
IL33	8.1
UHRF1	7.4
HIST1H3F	7.3
CLSPN	7.1
PSAT1	7.1
EXO1	6.5
PSAT1	*6.3*
PLEK2	6.1
IL13RA2	6.1
ID1	6.0
ZNF367	5.8
PRG4	5.7
TCF19	5.4
GPAM	5.4
C4orf26	5.4
GPR183	5.4
ASNS	5.4
IL11	5.3
HIST1H3I	5.3
SERPINE1	5.2
L2DTL	5.1

Gingival fibroblasts were treated with and without PRF lysates for 24 hours and a differential analysis was carried out.

Gene Ontology (GO) Enrichment Analysis of PRF lysates included biological process, molecular function, cellular component, KEGG (Kyoto Encyclopedia of Genes and Genomes) and Reactome. For example, Reactome enriched HSA-168249 (Innate Immune System), HSA-168256 (Immune System), HSA-109582 (Hemostasis), HSA-6798695 (Neutrophil degranulation), HSA-76002 (Platelet activation, signaling and aggregation) and HSA-114608 (Platelet degranulation). Top ranked in KEPP were hsa04610 (Complement and coagulation cascades) and hsa04611 (Platelet activation). Overall, the GO analysis of PRF lysates identified the expected activation of platelets and neutrophils but not explicitly the TGF-β-related enrichments. Thus, the question arises; What is the cellular response of gingival fibroblasts exposed to PRF lysates?

### Genomic profiling triggered by PRF lysates

To gain insights into the major response of gingival fibroblasts to PRF lysates, we analyzed the gene expression profile by genomics. To this end, gingival fibroblasts were either treated with PRF lysates for 24 hours or left untreated. The data revealed a total of 51 genes that were differentially expressed with at least 5-fold change. Among these genes, 28 were up-regulated (Table 1) and 23 were down-regulated (Table 2). A basic Gene Ontology Enrichment Analysis of genes regulated by PRF lysates was performed. For example, Molecular Function highlights GO:0005125 (cytokine activity), and Biological Processes that are activated are enriched for GO:0019221 (cytokine-mediated signaling pathway) and GO:0050896 (response to stimulus). Reactome highlights HSA-1280215 (Cytokine Signalling in Immune system) and HSA-913531 (Interferon Signaling). There was no explicit enrichment for NOX4, IL11, and PRG4. Nevertheless, NOX4 was the most strongly regulated gene by PRF lysates (Table 1) and consistent with our previous research on TGF-β signalling^23,25,26^, IL11 and PRG4 were also identified (Table 1). Thus, and based on our previous experience with enamel matrix derivative^25^, bone conditioned medium^26^ and acid bone lysates^23^, this triad of genes was selected to measure the response of fibroblasts to TGF-β activity.

**Table 2:** Down-regulated genes with at least 5x changes in oral fibroblasts exposed to PRF lysates.

Gene Symbol	Fold Change
IFI44L	0.09
MX1	0.09
CXCL10	0.09
MX2	0.10
OAS1	0.10
XAF1	0.13
SLC7A14	0.14
HERC5	0.15
LOC728613	0.15
DIRAS3	0.16
TSPAN2	0.17
LOC284412	0.18
TRABD2B	0.18
RORB	0.18
IL34	0.18
CYP7B1	0.18
HERC6	0.19
ADAMTS9-AS2	0.19
VCAM1	0.19
TNFSF13B	0.19
TGFB3	0.19
ALDH3A1	0.19
SLC39A8	0.19

Gingival fibroblasts were treated with and without PRF lysates for 24 hours and a differential analysis was carried out.

### PRF activates TGF-β signalling in human fibroblasts

**Figure 1:**
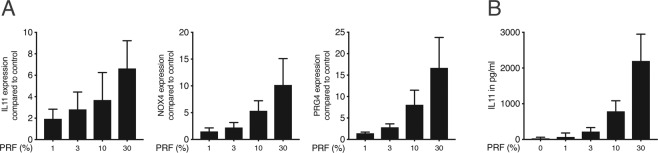
*PRF effect is dose-dependent*. Gingival fibroblasts were incubated with various concentrations of soluble extracts of PRF. (**A**) Reverse transcription PCR analysis for IL11, NOX4 and PRG4. (**B**) Quantification of IL11 in the supernatant by immunoassay. N = 3–4. Data are presented as mean ± SD.

To determine the most suitable concentration to produce significant changes on mRNA transcripts and proteins, gingival fibroblasts were exposed to different concentrations of PRF lysates. PRF acted in a dose-dependent manner showing that 30% of PRF was sufficient to induce a considerable increase of IL11, NOX4, and PRG4 (Fig. 1A). In line with mRNA transcripts, the dose-dependent activity was validated by IL11 immunoassay (Fig. 1B). These results indicate that 30% of PRF lysates can provoke a robust expression of IL11, NOX4, and PRG4 in gingival fibroblasts.

**Figure 2:**
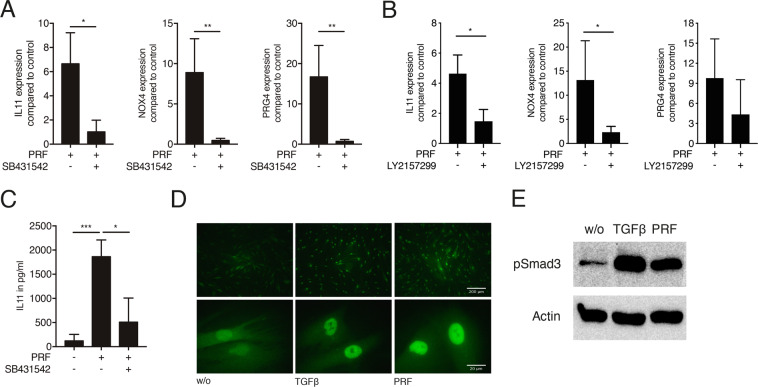
*PRF activates TGF-β signalling in human fibroblasts*. Gingival fibroblasts were stimulated with soluble PRF lysates in the absence or presence of the TGF- β receptor I kinase antagonist SB431542 or TGF- β receptor I inhibitor galunisertib LY2157299. (**A**) Reverse transcription PCR analysis for IL11, NOX4 and PRG4 with or without SB431542. (**B**) Reverse transcription PCR analysis for IL11, NOX4 and PRG4 with or without LY2157299. (**C**) Quantification of IL11 levels by immunoassay. (**D**) Representative immunofluorescence of the translocation of Smad2/3 into the nucleus upon stimulation with PRF and recombinant TGF-β, w/o; without. (**E**) Incubation of gingival fibroblasts with PRF increased phosphorylation of Smad3, treatment with 10 ng/ml of TGF-β was used as a positive control, full-length blots are presented in supplementary figure 2. N = 4–6. Data represent the mean ± SD, *P < 0.05, **P < 0.01, ***P < 0.001, by two-tailed Mann-Whitney test.

Considering the presence of TGF-β in PRF lysates and the robust up-regulation induced, we then sought to confirm whether TGF**-**β signalling mediates the up-regulation of the selected genes. To this end, the TGF-β receptor I kinase antagonist SB431542 and TGF-β receptor kinase inhibitor LY2157299 were utilized. Notably, SB431542 inhibited the up-regulation of all target genes (Fig. 2A). This inhibition was also confirmed with LY2157299, obtaining similar results (Fig. 2B). Moreover, SB431542 blocked the IL11 production on the protein level (Fig. 2B). As the canonical TGF-β activity acts via Smad proteins, we examined whether PRF triggers the nuclear translocation and the phosphorylation of smad3. Immunofluorescence analysis revealed that PRF promotes the nuclear translocation of Smad2/3 in gingival fibroblasts (Fig. 2C). Western blot analysis confirmed phosphorylation of smad3 by PRF (Fig. 2D). Taken together these findings indicate that IL11, NOX4, and PRG4 are the major triggers of PRF-derived TGF-β in gingival fibroblasts.

### Titanium surface and collagen membrane absorbs the PRF-derived TGF-β activity

**Figure 3:**
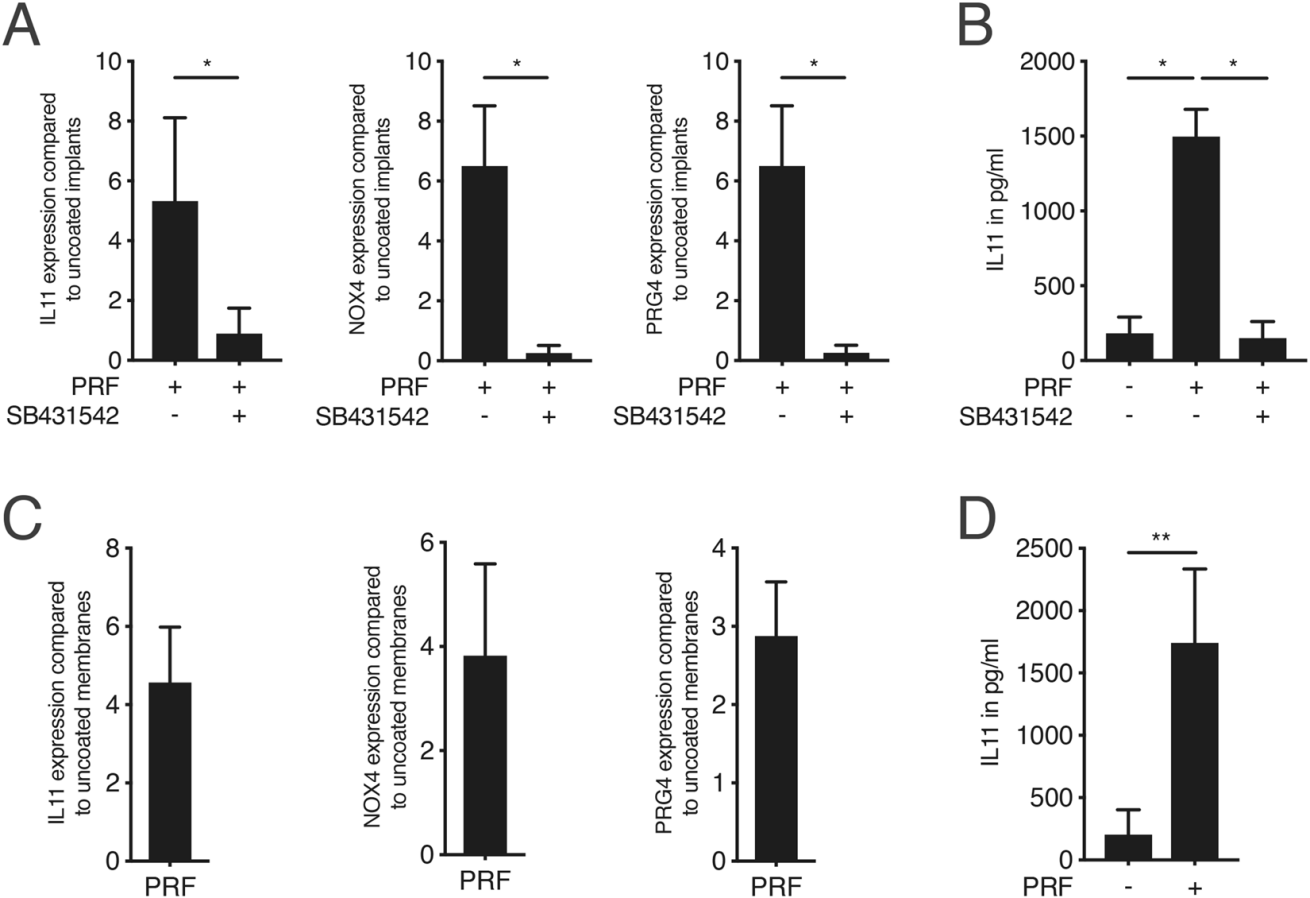
*PRF-derived TGF-β activity absorbs to titanium surface and collagen membrane*. Titanium discs were treated with PRF for 1 hour followed by three vigorous washes with buffered saline. Then, Gingival fibroblasts were seeded onto the PRF coated titanium discs overnight with and without the inhibitor for the TGF-β RI kinase SB431542. (**A**) Reverse transcription PCR analysis for IL11, NOX4 and PRG4. (**B**) Quantification of IL11 levels by immunoassay. Collagen membranes were treated with PRF lysates for 1 hour followed by three vigorous washes with buffered saline. Then, gingival fibroblasts were seeded onto the PRF-coated collagen membranes overnight. (**C**) Reverse transcription PCR analysis for IL11, NOX4 and PRG4. (**D**) Quantification of IL11 levels by immunoassay. N = 4–6. Data represent the mean ± SD, *P < 0.05, **P < 0.01, by two-tailed Mann-Whitney test.

Since PRF has been widely used during implant placement to boost osseointegration^5^ we examined first whether the PRF-activity absorbs to titanium surfaces. Gingival fibroblasts were seeded onto titanium discs as previously described^20^. Titanium surfaces exposed to PRF followed by vigorous washings with buffered saline caused a robust gene expression of IL11, NOX4, and PRG4 in gingival fibroblasts (Fig. 3A). As anticipated, SB431542 blocked the differential expression of the mRNA transcripts. To further verify this activity at the protein level, IL11 immunoassay was performed. PRF induced the release of IL11 into the supernatant of gingival fibroblasts and the presence of SB431542 significantly reduced its release (Fig. 3B).

PRF membranes have been increasingly applied in guided bone regeneration together with the standard collagen membrane to enhance bone regeneration^27^. As collagen can efficiently adsorb TGF-β^28^ we examined next whether the PRF-derived TGF-β activity absorbs to collagen membranes. Collagen membranes were exposed to PRF followed by vigorous washing with buffered saline. Then, gingival fibroblasts were seeded onto collagen membranes. Collagen absorbed the TGF-β activity of PRF lysates indicated by the differential expression of mRNA transcripts IL11, NOX4 and PRG4 (Fig. 3C) – and particularly by the strong increase of IL11 release into the supernatant (Fig. 3D). Altogether, these observations indicate that PRF-derived TGF-β activity absorbs to biomaterials such as titanium and collagen membranes.

### PRF-derived TGF-β activity is preserved at drilling temperatures

**Figure 4:**
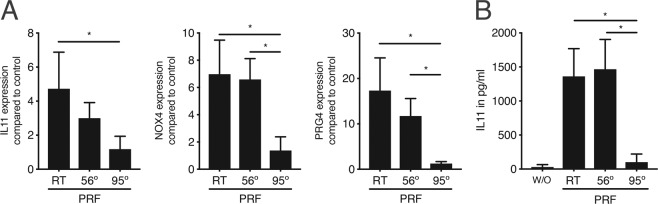
*PRF loses its TGF-β activity at high temperatures*. PRF lysates were heated either at 56° or 95°. Then, gingival fibroblasts were stimulated with unheated (RT) or heated PRF. (**A**) Reverse transcription PCR analysis for IL11, NOX4 and PRG4 using unheated or heated PRF. (**B**) Quantification of IL11 levels by immunoassay. w/o; without. N = 4–6. Data represent the mean ± SD, *P < 0.05, by two-tailed Mann-Whitney test.

PRF is commonly utilized as a coating agent for dental implants. Considering that during the drilling of the bone the temperature could increase above 47 °C^29^ and latent TGF-β can be thermally activated but also denaturated at high temperatures, we examined whether the temperature had an impact on the PRF activity. To this end, PRF extracts were heated either to 56° or 95°. Then, gingival fibroblasts were stimulated with heated or unheated PRF. The heat-treatment of PRF at 56 °C for five minutes did not affect the expression of the target genes relative to control PRF at room temperature (Fig. 4A). The heat-treatment of PRF at 95 °C for five minutes, however, abolished the TGF-β activity (Fig. 4A). These results were verified at the protein level via IL11 immunoassay (Fig. 4B). Our observations suggest that PRF-derived TGF-β activity is not affected significantly by the surgical procedure itself.

### PRF lysates reduce osteoblast differentiation in calvaria-derived osteoblasts

**Figure 5:**
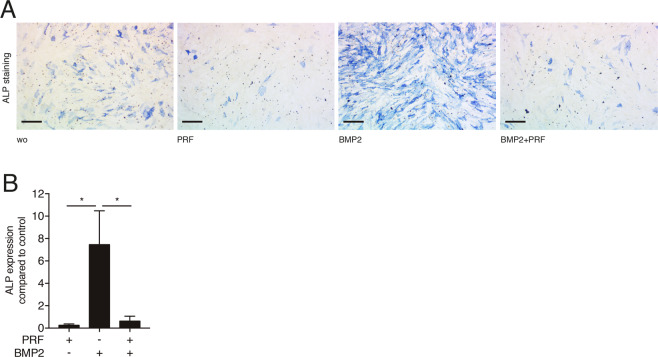
*PRF lysates attenuate osteoblast differentiation in calvaria-derived osteoblasts*. (**A**) Qualitative visualization of alkaline phosphatase staining in calvaria-derived osteoblasts. Scale bar is 500 μm. (**B**) Reverse transcription PCR analysis for ALP in the absence or presence of BMP2. BMP2 was used as a positive control. N = 4. Data represent the mean ± SD, *P < 0.05, by two-tailed Mann-Whitney test.

Apart from the triade of target genes identified, we performed an initial experiment to evaluate the impact of PRF lysates on osteogenic differentiation, a process that is essential for bone formation. We selected the widely used calvaria cells isolated from neonatal mice as a resource for osteogenic cells for *in vitro* assays^30^. Considering that recombinant TGF-β reduces alkaline phosphatase activity of murine calvaria-derived osteoblasts^31^, and that alkaline phosphatase is an early indicator of osteogenic differentiation^32^, we examined the effect of PRF lysates on the alkaline phosphatase activity on murine calvaria-derived osteoblasts. Notably, PRF reduced alkaline phosphatase activity as compared to the control group (Fig. 5A). This reduction was further verified by combining PRF with BMP2. These observations were corroborated at the gene expression level (Fig. 5B). Altogether, these findings further support the TGF-β activity of PRF lysates.

## Discussion

This is the first study on PRF that integrates robust and comprehensive analytical tools thereby providing insights into the cellular and molecular mechanisms triggered by PRF. One major finding was that among the 652 proteins identified in lysates of PRF membranes, TGF-β was one of the few growth factors found. Consistently, gene array revealed three TGF-β target genes namely IL11, NOX4, and PRG4 among the 51 strongly regulated genes by PRF lysates. Apart from inducing a robust TGF-β response in fibroblasts, these three target genes mediate downstream effects of TGF-β and play a role in bone homeostasis. For example, PRG4 regulates the maturation of the subchondral bone and holds an anti-inflammatory activity in synovial fibroblasts^33^. IL11 induces bone regeneration^34^ and acts synergistically with BMP-2 to enhance bone formation^35^. IL11 produced by fibroblasts is the dominant transcriptional response to TGFβ1 exposure and vital for its pro-fibrotic effect *in vivo*^36^. Lastly, NOX4 is related to the activation of bone-resorbing osteoclasts^37^ and also mediates the TGFβ1-driven myofibroblasts differentiation in lung fibrosis^38^. Therefore, our findings also raise the possibility that IL11 and NOX4, in response to PRF lysates, may induce a pro-fibrotic differentiation in fibroblasts.

If we relate the current findings to those described by others, our proteomics approach confirms the work based on traditional immunoassays showing the release of TGF-β among other growth factors from PRF membranes^3,17^. These results mirror those observed with previous preparations with leucocyte-depleted platelets subjected to proteomic analysis^18,39^. Our research strategy is in line with gene array approaches to identify major target genes, for example in gingival fibroblasts exposed to enamel matrix derivative^25^, acid bone lysates^23^, and PRP gene arrays in a mouse model of mandibular advancement^40^. It is thus not surprising that the TGF-β activity of PRF lysates is a major pathway indicated by the robust up-regulation of IL11, NOX4 and PRG4. This was confirmed by using two independent TGF-β receptor kinase inhibitors thereby blocking the expression of IL11, NOX4 and PRG4. The activation of the Smad2/3 canonical signalling pathway by PRF corroborated our findings, which are in line with previous research on bone-derived TGF-β^20,23^. However, it is unlikely that TGF-β is the only growth factor in PRF lysates capable of inducing a cellular response. Indeed, the platelet lipidome comprises almost 400 lipid species^41^ such as the potent sphingosine-1-phosphate affecting bone turnover^42^ which are not covered by the proteomic analysis.

The fact that other classical growth factors such as PDGF isoforms, VEGF and or IGF1^17^ were not identified by proteomic analysis does not fully discard their presence in the sample since they might be below the limit of detection or simply render few detectable peptides. Growth factors and cytokines are usually found in low concentrations in biological samples but, even in such low concentrations, they are able to display their functions. We also identified HDGF that plays a pro-fibrogenic role during liver fibrosis in mice through activation of TGF-β pathway^43^ and MYDGF that helps to protect and repair the heart after acute myocardial infarction^44^. We further found PF4, also known as chemokine ligand 4 (CXCL4), which is released from alpha-granules of activated platelets^45^. Nonetheless, the impact of HDGF, MYDGF and PF4 on gene expression changes in oral fibroblasts remains to be investigated. As a consequence, we mainly focused on the three strongly regulated genes that we have previously identified to be sensitive to TGF-β signalling^23,25,26^. Further research should therefore identify the other signaling pathways that lead to the differentially gene expression in response to PRF lysates. We are currently preparing a genomic analysis similar to our previous approach on enamel matrix derivatives^25^ with and without the presence of the TGF-β receptor I kinase antagonist SB431542 in order to differentiate the genes targeted by TGF-β and those regulated independently this signaling pathway.

To gain further insights into the biological effect of PRF, we investigated the osteoblastic differentiation based on alkaline phosphatase histochemical staining and gene expression analysis in calvaria cells. Alkaline phosphatase activity is an early osteoblast differentiation marker^32^. We found here that PRF lysates attenuated the alkaline phosphatase activity. PRF lysates could even reduce the BMP2-induced up-regulation of the alkaline phosphatase. These observations are in alignment with a previous study on periodontal ligament fibroblasts^46^ and might be attributed to the PRF-derived TGF-β activity thereby reducing the alkaline phosphatase activity on calvaria-derived osteoblasts^31^. The role of TGF-β signalling to mediate the effects of PRF lysates on osteogenic differentiation and bone formation, however, remains to be determined. In contrast, other studies have found an enhancement of alkaline phosphatase by PRF in many cell types^47^, including human bone cells^13^, mesenchymal cells^12^ and pulp cells^48^. These conflicting results might be explained to some extent by the methodological discrepancies and the different cell types. The majority of the aforementioned reports used different protocols and centrifuges to produce PRF^17^.

Although the clinical relevance of our discoveries is a matter of speculation, they might have an impact on guided bone regeneration (GBR) and implant dentistry. GBR is a common biomaterial-based and well-documented clinical procedure that aims at the regeneration of hard tissues either in alveolar bone defects or in peri-implant defects^49^. GBR is based on the principle of using a barrier membrane with a bone graft underneath. Recently, this procedure has been combined with PRF in order to maximize the clinical outcomes^9^. Even though the incorporation of growth factors into collagen membrane is not novel we are the first to show that PRF-derived TGF-β activity adsorbs to collagen membrane and resists vigorous washings. The present findings were not unexpected since TGF-β, BMP2 and other growth factors can adsorb to collagen^21,22^ and are combined for better growth factor binding and release. Furthermore, we showed that PRF-derived TGF-β activity also adsorbs to titanium surfaces. This accords with our earlier observations, which showed that the bone-derived TGF-β activity binds to titanium surfaces^20^. Considering that PRF increases implant stability of dental implants at early stages^5,8^, PRF-derived TGF-β activity may play a critical role in the early phases of osseointegration^20^. In this sense, a sustained delivery of growth factors by functionalized biomaterials, particular collagen membranes and dental implants, might be advantageous for bone regeneration and wound healing, nonetheless future research is necessary to confirm this hypothesis.

The present study has a number of limitations. First, considering the debates on the impact of g-forces and the selection of tubes on the composition of PRF^50^, we only applied  one protocol, using 400 g calculated at RCF-max to prepare PRF membranes. Second, the screening for target genes is based on a selection of three highly expressed genes that were identified downstream upon TGF-β receptor activation mainly based on our previous research with enamel matrix derivative^25^, acid bone lysates^23^, and bone conditioned medium^26^. Hence, other genes that are also responsive to PRF playing a possible role *in vivo* might have been neglected. Third, gingival fibroblasts where not characterized and after a few passages the cells may change their expression pattern demanding further characterization. However, our data are rather consistent with previous reports. Furthermore, gingival fibroblasts represent one out of many possible target cells for PRF and it is likely that the beneficial effect of PRF *in vivo* requires the crosstalk between multiple cell types. The robust TGF-β activation might only be relevant for mesenchymal cells while other cell types require the activation by other cues apart from growth factors. Finally, even though we have identified the adsorption of the TGF-β activity to titanium and collagen membranes, and both biomaterials are routinely used for oral regeneration^8,27,51^, the clinical implication of these findings remains unclear but inspires future research.

Future research should therefore focus on other cell types to further evaluate the potential of tissue-targeted PRF. Moreover, owing to the diversity of PRF protocols across the disciplines, further research is necessary for detailed constituents of different PRF preparations. It remains unclear whether TGF-β plays a pivotal role in all the different preparations. Our study provides, however, a valuable PRF dataset for future comparisons especially in terms of protein expression profiles. The interaction and combination of different biomaterials with PRF will require tight control and understanding of the host system to ensure cell engraftment and tissue responsiveness. The most suitable combination to program cell fate resulting in predictable therapeutic outcomes, still remains to be elucidated. There is a clinical need for predictable therapeutic strategies that can translate into everyday use, not only in clinical dental practice but also across other medical specialties. In this sense, PRF offers us a unique opportunity in clinical translational research since autologous preparations of growth factors from blood usually need no formal approval thereby accelerating the translation of this therapy.

## Conclusion

In summary, the present study revealed that the expression of the TGFβ target genes IL11, NOX4 and PRG4 on gingival fibroblasts is a dominant transcriptional response to PRF.

## Material and Methods

### Preparation of PRF lysates and conditioned medium

PRF membranes were obtained and prepared following a standard protocol previously reported^52,53^. After the approval by the ethics committee of the Medical University of Vienna (1644/2018), venous blood was collected at the University Clinic of Dentistry from six healthy volunteers. Before drawing the blood, each volunteer was required to sign an informed consent to participate in the study. Then, each volunteer donated six plastic glass-coated tubes (BD Vacutainer® Ref 367896; BD, Plymouth, UK) allowing spontaneous blood coagulation. Platelet Rich Fibrin (PRF) clots were produced utilizing a protocol of 1570 RPM for 12 minutes (RCF-max = 400 g). PRF membranes were produced utilizing a centrifuge device (Z 306 Hermle Universal Centrifuge, Wehingen, Germany) with universal swing-out rotors (146 mm at the max) utilizing 10 mL glass-coated plastic tubes. The PRF clot was separated from the remaining red thrombus and compressed between two layers of dry gauze. Each PRF membrane is transferred into serum-free medium (1 cm PRF/ml) and exposed to two cycles of freeze-thawing and sonication (Sonopuls 2000.2, Bandelin electronic, Berlin, Germany) as reported for human platelet lysate^15,54,55^. After centrifugation (Eppendorf AG-22331, centrifuge 5424, Hamburg, Germany) at 15000 g for 10 minutes, the supernatants of the PRF membranes were harvested and stored at −20 °C prior to the *in vitro* analysis. In indicated experiments, PRF membranes were transferred into serum-free medium (1 cm PRF/ml) and placed into an incubator at 37 °C to allow a natural release of growth factors into the culture media, similarly as previously described^17^. At 24 hours the conditioned medium was collected. All experiments were performed in accordance with relevant guidelines and regulations.

### Cell culture

Tissue samples of human gingiva were harvested from extracted third molars from patients who had given informed and written consent. Before obtaining the samples, the Ethics Committee of the Medical University of Vienna (EK NR 631/2007) approved the protocol. A total of three strains of fibroblasts were established by explant cultures and fewer than 10 passages were used for the experiments. Calvaria-derived osteoblasts were obtained according to a standard protocol previously described (Oishi *et al*. 2016). Briefly, mouse pups less than 5 days old were euthanized and their calvaria collagenase digested through a series of sequential digestions. The first 2 digests were discarded, and the subsequent digests were pooled and plated. Cells were seeded at a concentration of 30,000 cells/cm² onto culture dishes one day prior to stimulation. Cells were also treated overnight with and without different concentrations of PRF lysates up to 30% in serum-free media before gene expression was analyzed. Alternatively, cells were stimulated with heated PRF at 56 °C or 95 °C. In indicated experiments, cells were exposed to PRF conditioned medium instead of PRF lysates. Additionally, machined titanium discs (Ti Gr. 4; Implacil De Bortoli, São Paulo, Brazil) and collagen membranes (Bio Guide®, Geistlich Biomaterials, Wolhusen, Switzerland) were immersed in PRF for one hour at room temperature, followed by three washings with phosphate buffer saline as previously described^20^. To examine the influence of TGF-β signaling, the inhibitor of TGF-β receptor type I kinase, SB431542 (Calbiochem, Merck, Billerica, MA) was used at 10 µM.

### Cell differentiation

As stated recently^23^, for osteogenic differentiation, calvaria cells were incubated in growth medium containing 50 μg/mL ascorbic acid (Sigma Aldrich, St. Louis, MO) and 10 mM beta glycerophosphate (Sigma Aldrich, St. Louis, MO) as previously reported^23^. Alkaline phosphatase staining was performed after 3 day. For histochemical staining of alkaline phosphatase, cells were fixed as indicated and incubated with a substrate solution containing naphthol AS-TR phosphate and fast blue BB salt (Sigma Aldrich, St. Louis, MO)^56^. After rinsing with distilled water, cultures were photographed. Slides were imaged using an Oxion Inverso microscope (Euromex, Arnhem, The Netherlands) with a 4× objective.

### Proteomic analysis

The detailed protocol is presented in the supplement and was recently reported^23^. PRF lysates from a pool of three independent donors were subjected to proteomic analysis. In brief, PRF lysates were dissolved in 1% Rapigest in 50 mM TEAB and the solution was filtered through a molecular-weight cut-off filter of 100 kDa first. The resulting filtrate was then passed through a 50 kDa filter and four fractions were generated – two filtrates and two concentrates. Extracted proteins were reconstituted from the membrane and from the filtrate, precipitated using methanol/dichloromethane and digested with trypsin as described earlier^57^. In total four fractions were generated and analyzed. Protein concentration was determined using the DeNovix DS-11 FX Spectrophotometer (Wilmington, USA) and proteins were reduced using 5 mM DTT (Dithiothreitol, Sigma-Aldrich, Vienna, Austria) for 30 minutes at 60 °C, and alkylated for 30 minutes with 15 mM IAA (Iodoacetamide, Sigma-Aldrich, Vienna, Austria) in the dark. Finally, porcine trypsin (Promega, Vienna, Austria) was added in a ratio trypsin to protein 1:50 (w/w). After 16 hours of incubation at 37 °C, aliquots of 20 µl were prepared and stored in 0.5 ml protein low-bind vials (Eppendorf, Vienna, Austria) at −20 °C until injection on next day. The nano HPLC separation of each fraction was performed using a nanoRSLC UltiMate 3000 HPLC system by Thermo Fisher. Raw MS/MS files were analyzed using Proteome Discoverer 2.2 (ThermoFisher Scientific, Bremen, Germany) and searching the Swissprot database (Homo sapiens, http://www.uniprot.org/proteomes/UP000009606, version from May 2019). Search parameters are presented in the Supplement material. All search results were refined and researched using Scaffold 4.6.5 (Proteome Software, Portland, OR). For the classification of the proteins the STRING online software was applied (https://string-db.org)^58^. The mass spectrometry proteomics data have been deposited to the ProteomeXchange Consortium (www.proteomexchange.org) via the PRIDE partner repository (www.ebi.ac.uk/pride) with the dataset identifier PXD014382 and 10.6019/PXD014382.

### Microarray analysis

Microarray analysis was carried out as previously described^23^. Total RNA from gingival fibroblasts exposed to 30% PRF lysates or left untreated was harvested with the RNA Isolation Kit (Extractme, BLIRT S.A., Gdańsk, Poland). RNA quality was determined using the Agilent 2100 Bioanalyzer (Agilent Technologies, Santa Clara, CA, USA). Microarray analysis was performed using the SurePrint G3 Human Gene Expression v2 Microarray (Agilent Technologies, Santa Clara, CA). Array image acquisition was performed with the Agilent G2505B Microarray Scanner and Feature Extraction software version 9.5 (Agilent). Background-corrected fluorescence intensity values were imported into GeneSpring v.15, log2-transformed, and then normalized by quantile normalization. A filtering step was applied in order to reduce the number of multiple hypotheses. Only genes for which at least 100% of the values in one of the two evaluated conditions were above the 60th percentile were used for further analysis. Differentially expressed mRNAs were identified by paired t-tests in GeneSpring. The resulting p-values were corrected for multiplicity by applying Benjamini-Hochberg adjustment to all p-values calculated for a time point with a false discovery rate (FDR)  <  5%^59^. Genes with an adjusted p-value <0.05 were considered significant. Shown are genes that are at least 5-fold changed compared to an unstimulated control.

### RT-PCR and immunoassay

Comparable to our previous research^23^, total RNA was isolated with the ExtractMe total RNA kit (Blirt S.A., Gdańsk, Poland). Reverse transcription (RT) was performed with the SensiFAST™ cDNA Synthesis Kit (Bioline Reagents Ltd., London, UK). RT-PCR was done with SensiFAST™ SYBR® Kit using manufacturer’s instructions (Bioline). Amplification was performed with the Biorad CFX Connect Real-Time PCR System (CFX Connect, BioRad, Hercules, CA, USA). Primer sequences are given in Table 3 except for IL11 which was purchased from BioRad (Hercules, CA, USA). Relative gene expression was calculated with the delta delta CT method using the analysis CFX Maestro™ software (BioRad, Hercules, CA, USA). Reactions were run in duplicates. The supernatant was analyzed for IL11 by immunoassay according to the manufacture’s instruction (R&D Systems, Minneapolis, MN, USA).

**Table 3:** Primer list.

	Sequence_F	Sequence_R
hPRG4	cag ttg cag gtg gca tct c	tcg tga ttc agc aag ttt cat c
hNOX4	tct tgg ctt acc tcc gag ga	ctc ctg gtt ctc ctg ctt gg
hGAPDH	aag cca cat cgc tca gac ac	gcc caa tac gac caa atc c
mALP	aac cca gac aca agc att cc	gag aca ttt tcc cgt tca cc
mbactin	cta agg cca acc gtg aaa ag	acc aga ggc ata cag gga ca
mGAPDH	aac ttt ggc att gtg gaa gg	gga tgc agg gat gat gtt ct

### Western blot

Western blot was conducted as reported^23^. In brief, cell extracts containing SDS buffer and protease inhibitors (PhosSTOP with cOmplete; Sigma, St. Louis, MO) were separated by SDS-PAGE and transferred onto PVDF membranes (Roche Diagnostics, Mannheim, Germany). Membranes were blocked and incubated with the first antibody (rabbit anti-pSmad3 Ser423/425, 1:500, Abcam, ab52903, Cambridge, UK) and actin (Santa Cruz Biotechnology, SCBT, Santa Cruz, CA, USA) overnight. The primary antibodies were then detected using the appropriate secondary antibody. Subsequently, chemiluminescence detection was performed with a ChemiDoc MP System (Bio‐Rad Laboratories, Inc. CA, USA).

### Immunofluorescence

Immunofluorescent analysis was performed as recently described^23^. Briefly, human gingival fibroblasts were plated onto Millicell® EZ slides (Merck KGaA, Darmstadt, Germany) and treated with PRF 30% for 30 min. Cells were then fixed in paraformaldehyde and blocked in 5% BSA and 0.3% Triton in PBS at room temperature for 1 hour. Cells were subsequently incubated with Smad2/3 antibody (1:800; D7G7 XP® Rabbit mAb #8685, Cell Signaling, MA, USA) overnight at 4 °C. Alexa Fluor 488 secondary antibody (1:1000; Anti-Rabbit, Cell signaling Technology, USA) was applied for 1 hour. Cells were washed and mounted onto glass slides. Fluorescent images were captured at 10x and 100x in oil immersion using a Zeiss Axiovert 200 M fluorescent microscope.

### Statistical analysis

The experiments were repeated three to five times. Bars show the mean and standard deviation of the cumulative data from all experiments. The Shapiro–Wilk test was used to test the normality of the data sets. Statistical analysis was based on Mann-Whitney U test and Kruskal-Wallis test with Dunn’s multiple comparisons correction. Analyses were performed using Prism v7 (GraphPad Software, La Jolla, CA). Significance was set at p < 0.05.

## Supplementary information

Supplementary Information.

Supplementary Data.

## Data Availability

The raw required to reproduce the findings from this study will be made available to interested investigators upon request.
