# Supplementary material for: TGFβ activity released from platelet-rich fibrin adsorbs to titanium surface and collagen membranes
Source: Sci Rep. 2020 Jun 23;10:10203. doi: 10.1038/s41598-020-67167-3 (PMC7311486; doi:10.1038/s41598-020-67167-3)
Supplement: Supplementary file 1 — Supplementary Information. [file 41598_2020_67167_MOESM1_ESM.pdf]

Supplementary Information

TGFβ activity released from platelet-rich fibrin adsorbs to titanium surface and collagen membranes

Francesca Di Summa, Zahra Kargarpour, Jila Nasirzade, Alexandra Stähli, Goran Mitulović, Tanja Panić-Janković, Veronika Koller, Cosima Kaltenbach, Heinz Müller, Layla Panahipour, Reinhard Gruber, Franz-Josef Strauss

Supplemental Methods. Mass spectrometry.
Supplementary Figure 1. Full-length blots, incubation of gingival fibroblasts with PRF caused an increased phosphorylation of Smad3.

Supplemental Methods.

Nano HPLC Separation and MS Analysis Methods

Mobile phases applied for sample loading, desalting, and separation were 0.01% aqueous heptafluorobutyric acid (HFBA) solution was used for sample loading applying a User Defined Program for sample injection. The loading mobile phase was delivered to the trap column at 30µl/min using the loading pump. Mobile phases for peptide separation on the nano separation column were: (i) A: 95% Acetonitrile (AcN), 5% Water, 0.1% Formic acid; (ii) B: 50% AcN, 30% Methanol (MeOH), 10% 2,2,2-Trifluoroethanol (TFE), 10% Water, 0.1% Formic acid (FA). (iii) Autosampler loading solvent for sample injection was 0.1% aqueous TFA, 0.01% HFBA, 2% can; (iv) Wash of the injection needle, sample injection valve, and the trap column was performed using 100% TFE.

Trapping column used for sample loading, concentration and clean-up was a C18 µPAC trap column, (PharmaFluidics, Gent, Belgium). Nano chromatographic separation of peptides was performed on a C18 µPAC (µ-Pillar-Arrayed-Column, PharmaFluidics, Gent, Belgium). The pillars had an interpillar distance of 2.5µm, and the total separation path was 2m. Both, the trap and the separation column were operated in a column oven at 50°C. Sample was loaded onto the trap column for 10 minutes when the valve switched the position and the nano gradient was directed through the trap column and onto the separation column. The trap column was switched back into the flow path of the loading column at 170 minutes in the runtime for equilibration and preparation of the following injection.

Separation was performed at 600nl/min and the gradient was formed as follows: An isocratic start with 2% B was maintained for 10 minutes and was followed by increasing the amount of B to 60% until 150 minutes. The column and the trap column were flushed with 90% B for 15 minutes, until 160 minutes, which was followed by equilibration of 25 minutes for the separation column. Blank samples (injection of loading solvent) were run between sample injections for cleaning the separation system, preventing carry-over and background control.
Prior to mass spectrometric detection and analysis, peptides were detected using the UV at 214 nm in a 3nl cell. Mass spectrometric detection and MS/MS analysis was performed using the Q-Exactive Plus Orbitrap BioPharma mass spectrometer (ThermoFisher, Bremen, Germany). Peptides were introduced into the nano electrospray

source (ESI) after the UV cell and the ionization was performed using the stainless steel needle with 20 µm inner diameter and 10µm tip. Needle voltage was set to 2.8 kV in positive mode and the top 10 ions were selected for MS/MS analysis (fragmentation), resolution was set to 70.000 for full MS scans, ions with single charge were excluded from MS/MS analysis and fragmented ions were excluded for 60 seconds from further fragmentation.

Data Analysis

Raw MS Data were searched against the human SwissProt protein database (Version June 2019) with Proteome Discoverer 2.4 (ThermoFisher Scientific, Bremen, Germany) using following parameters:

- Taxonomy: Homo sapiens
- Modifications: carbamidomethyl on C as fixed, carboxymethylation on M as variable
- Peptide tolerance was set to 10 ppm and the MS/MS tolerance to 0.05Da
- Trypsin was selected as the enzyme used and two missed cleavages were allowed
- False discovery rate (FDR) was set to 1% and the decoy database search was used for estimating the FDR.*Data Analysis*

Raw MS Data were searched against the human SwissProt protein database (Version June 2019) with Proteome Discoverer 2.4 (ThermoFisher Scientific, Bremen, Germany) using following parameters:

- Taxonomy: Homo sapiens
- Modifications: carbamidomethyl on C as fixed, carboxymethylation on M as variable
- Peptide tolerance was set to 10 ppm and the MS/MS tolerance to 0.05Da
- Trypsin was selected as the enzyme used and two missed cleavages were allowed
- False discovery rate (FDR) was set to 1% and the decoy database search was used for estimating the FDR.

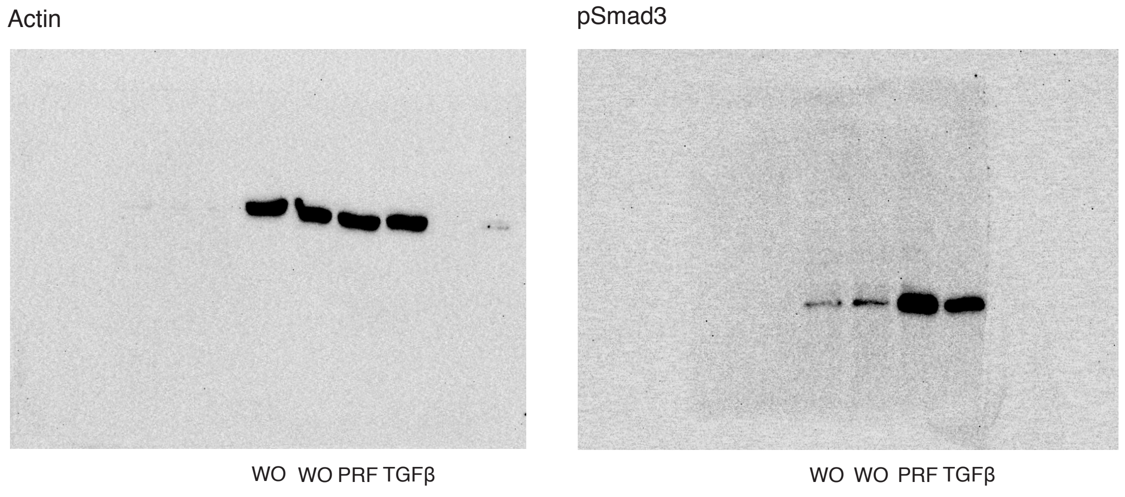

Supplementary Figure 1. Full-length blots, incubation of gingival fibroblasts with PRF lysates caused an increased phosphorylation of Smad3.
